# Dysregulation of oxytocin and dopamine in the corticostriatal circuitry in bipolar II disorder

**DOI:** 10.1038/s41398-020-00972-6

**Published:** 2020-08-12

**Authors:** Shyh-Yuh Wei, Huai-Hsuan Tseng, Hui Hua Chang, Tsung-Hua Lu, Wei Hung Chang, Nan Tsing Chiu, Yen Kuang Yang, Po See Chen

**Affiliations:** 1 Department of Psychiatry, National Cheng Kung University Hospital, College of Medicine, National Cheng Kung University, Tainan, Taiwan; 2 Institute of Behavioral Medicine, College of Medicine, National Cheng Kung University, Tainan, Taiwan; 3 Institute of Clinical Pharmacy and Pharmaceutical Sciences, College of Medicine, National Cheng Kung University, Tainan, Taiwan; 4 School of Pharmacy, College of Medicine, National Cheng Kung University, Tainan, Taiwan; 5 Department of Pharmacy, National Cheng Kung University Hospital, College of Medicine, National Cheng Kung University, Tainan, Taiwan; 6 Department of Pharmacy, National Cheng Kung University Hospital, Dou-Liou Branch, Yunlin, Taiwan; 7 Department of Psychiatry, National Cheng Kung University Hospital, Dou-Liou Branch, Yunlin, Taiwan; 8 Department of Nuclear Medicine, National Cheng Kung University Hospital, College of Medicine, National Cheng Kung University, Tainan, Taiwan; 9 Department of Psychiatry, Tainan Hospital, Ministry of Health and Welfare, Tainan, Taiwan

**Keywords:** Bipolar disorder, Molecular neuroscience

## Abstract

The oxytocin (OXT) and dopamine systems synergistically facilitate striatal reactivity. Abnormal striatal activation has repeatedly been observed in patients with bipolar disorder (BD); however, such abnormality remains unclear in BD II. Here we aimed to investigate whether the corticostriatal connectivity was altered and the possible relationships among corticostriatal connectivity, OXT, and dopamine systems in BD II. Twenty-five BD II patients, as defined by the DSM-V, and 29 healthy controls (HC) were enrolled in this study. Plasma OXT was measured and striatal dopamine transporter (DAT) availability was assessed using [^99m^Tc]TRODAT-1 single-photon emission computed tomography (SPECT). Brain network functional connectivity (FC) was measured during the resting-state using functional magnetic resonance imaging, and the dorsal caudate (DC) was selected as the seed region. The results showed that the OXT level was significantly lower in the BD II patients, while the striatal DAT availability was not significantly different between the BD II and HC groups. The BD II patients exhibited significantly lower FC between the DC and the executive control network (dorsolateral prefrontal, anterior cingulate cortex, and posterior parietal cortex) as compared with the HC. Only observed in HC, the DC-posterior parietal cortex FC was negatively correlated with the OXT level and striatal DAT availability. Our findings in the HC support a model in which the OXT and dopamine systems act in tandem to regulate corticostriatal circuitry, while the synergistic interaction was perturbed in BD II. Taken together, these results implied a maladaptive neuroplasticity in BD II.

## Introduction

The oxytocin (OXT) and dopamine systems interact and synergistically facilitate striatal reactivity for proper social behaviors^1^. Both social behaviors and the corticostriatal circuitry are mediated by OXT^2,3^ and dopamine^4^. Oxytocinergic neurons are projected from the hypothalamus to both the ventral tegmental area and substantia nigra^1^, while dopaminergic neurons are projected from the ventral tegmental area and substantia nigra to the ventral and dorsal areas of the striatum^5^. The dorsal striatum is involved in the reward system, reactive to social stimuli, as social behaviors may be differentiated by the corticostriatal functional connectivity (FC) strength in animals^6^ or by the caudate FC patterns in humans^7^.

The caudate is one of the most important neural substrates of the corticostriatal circuitry and is associated with cognition^8^. The projections from the prefrontal (associative circuit) and anterior cingulate (limbic circuit) cortex terminate in the caudate, then the caudate projects to other parts of the basal ganglia and back to the cortex via the thalamus^8,9^. Such corticostriatal circuitry is essential in motivating behaviors, and its dysfunction is well characterized in neuropsychiatric disorders^10,11^, including bipolar disorder (BD)^10^.

Previous neuroimaging studies have unraveled neuroplastic changes in the corticostriatal circuitry of patients with BD in terms of structural volume^10^, intrinsic FC^12^, and activity that may influence emotional processing^13^. Furthermore, altered FC between the caudate and default mode network (DMN)^14^ and between the caudate and executive control network (ECN)^15,16^ has been observed in BD patients as compared with controls. The ECN involves the dorsolateral prefrontal cortex (dlPFC), anterior cingulate cortex (ACC), and posterior parietal cortex (PPC)^17^. Different activation patterns in these networks may contribute to mood dysregulation^18^ and may distinguish BD from unipolar depression^15,19^. However, these patterns remain elusive in patients with BD II, because previous studies did not include BD II patients or distinguish them from BD I patients, even though BD II patients are as disabled as BD I patients^20,21^. In the current study, we aimed to investigate whether the caudate-seeded connectivity was altered in the DMN and ECN in BD II.

The dopamine hypothesis is a key theory in BD^22^, and BD is associated with abnormal dopamine transporter (DAT) levels^23^ and genetic factors of DAT^24^. As striatal dopamine deficits predict reductions in striatal FC in major depression^25^, we aimed to explore the relationships between possible FC alterations and striatal DAT in BD II. In addition, we have previously reported that BD II patients exhibited significant dysregulation in the plasma OXT level^26^. As the corticostriatal circuitry is mediated by OXT^2,3^ and dopamine^4^, we also aimed to explore the association between corticostriatal FC and OXT in BD II.

## Methods

### Subjects

All patients were recruited from the psychiatric outpatient department at National Cheng Kung University Hospital, while all healthy controls (HC) were recruited from the community through advertisement. All participants, aged between 18 and 70 years, were recruited and evaluated by an attending psychiatrist using the Chinese version of the Mini International Neuropsychiatry Interview^27^, the 17-item Hamilton Depression Rating Scale (HDRS) and the 11-item Young Mania Rating Scale (YMRS).

All patients were diagnosed by a psychiatrist to determine eligibility according to the Diagnostic and Statistical Manual of Mental Disorders, Fifth Edition (DSM-V). The exclusion criteria for all the participants were as follows: (1) major mental illnesses (other than BD II for the BD II patients); (2) a history of head trauma, organic mental disease, or other neurological disorders; (3) inflammatory diseases, serious surgical conditions, or severe physical illnesses, such as acute coronary syndrome, kidney dialysis, or transplant; and (4) plans for pregnancy, breastfeeding, or a positive pregnancy test.

The study was conducted in accordance with the Declaration of Helsinki and was approved by the Institutional Review Board of National Cheng Kung University Hospital. All participants provided their written informed consent.

### Experimental design

After enrollment into this study, the administration of medications, such as mood stabilizers (i.e., valproic acid or lithium), was recorded and adjusted according to the clinical manifestation and patients’ tolerance. There was no main regimen adjustment during the study.

### Levels of plasma oxytocin

Blood samples for the OXT assay were collected in 5-mL EDTA tubes and stored at 4 °C in a fridge. Plasma was isolated by centrifugation at 1800×*g* for 15 min at 4 °C and immediately stored at ‒80 °C. OXT immunoreactivity levels were quantified in duplicate using a commercial OXT ELISA kit (Enzo Life Sciences, NY, USA, formerly Assays Designs, MI, USA). The detection range was from 12.35 to 1000 pg/ml, while the sensitivity (i.e., the minimum detectable dose of OXT) of our assay was <4.92 pg/ml. There was no extraction. The intra-assay precision and inter-assay precision of the assay were <10 and 12%, respectively (coefficient of variance (c.v.) (%) = SD/mean × 100; intra-assay: c.v. <10%; inter-assay: c.v. <12%).

### [^99m^Tc]TRODAT-1 single-photon emission computed tomography (SPECT)

The imaging procedure was identical to that used in our previous study. For detailed information regarding the selective labeling of DAT and obtaining/reconstructing/co-registering SPECT images, please refer to our published papers^28–30^. The MRI images were used as a reference, and so the slice thickness of the co-registered images was the thickness of the T2-weighted MRI images (3.3 mm). Regions of interest were the striatum (basal ganglia, caudate nucleus, and putamen) and occipital cortex, and the ratio of the radioactivity [(St – Oc)/Oc ratio] was then derived by dividing the difference between the average activity in the striatum (St) and the average activity in the occipital cortex (Oc) by the average activity in the occipital cortex (Oc)^31^.

### Image acquisition

Resting-state functional MRI images were acquired using a 3.0 Tesla MRI scanner (MR750, GE Medical Systems, Milwaukee, WI, USA) with an 8-channel head coil in the Mind Research and Imaging Center of National Cheng Kung University. High-resolution T1-weighted 3-dimensional structural images ([TR]/[TE] = 7.7 ms/2.9 ms, flip angle = 12°, field-of-view = 224 mm^2^, in-plane matrix size = 256 × 256, slice thickness = 1 mm, and slices = 166) and T2*-weighted echoplanar imaging sequences ([TR]/[TE] = 2000 ms/33 ms, flip angle = 90°, field-of-view = 240 mm^2^, in-plane matrix size = 64 × 64, slice thickness = 3 mm, and slices = 40) were conducted to obtain high-resolution anatomical T1 images and functional MRI images. The first five functional scans of each resting-state functional MRI series were discarded for signal saturation and magnetic field stabilization. The participants remained awake during the scan (eyes closed, head still but relaxed, and without thinking about anything in particular). Head cushions and earplugs were provided to reduce head motion and noise, respectively.

### Image preprocessing

Preprocessing was performed using the DPARSF toolbox (State Key Laboratory of Cognitive Neuroscience and Learning, Beijing Normal University, China) with Statistical Parametrical Mapping 12 (SPM 12, Wellcome Trust Centre for Neuroimaging, London, http://www.fil.ion.ucl.ac.uk/spm) in MATLAB 2016a (MathWorks Inc., Natick, MA USA). All functional images were subjected to slice timing, realignment for head-motion correction, and co-registration against each individual’s anatomical image, as well as normalization against the International Consortium for Brain Mapping (ICBM) space template of East Asian brains. Subjects with head motion of any volume >2 mm or 2° were excluded from further processing. The images were re-sampled to an isotropic 2 mm^3^ voxel size during the normalization step and then spatially smoothed using a 3D Gaussian kernel of 6 mm full-width at half-maximum. Linear trends were then removed from the resulting time series, and the time series was temporally band-pass filtered (0.01–0.1 Hz) in order to extract the low-frequency oscillations associated with spontaneous neuronal activity.

### Definition of caudate seed and caudate-seeded functional connectivity maps

The left and right dorsal caudate (DC) seeds (3 mm radius), centered at MNI coordinates (±13, 15, and 9), were identified based on the published literature^32^. The mean time-series activity in the seed region of each subject was extracted. The DC-seeded FC maps were then generated. Each individual-level FC map obtained was then converted into a z-map using Fisher’s r-to-z transformation for second-level group analyses.

### Additional test to examine specificity

One potential concern was whether the results were generic across the whole basal ganglia or specific to the DC. To further address this concern, we performed identical functional analyses using seeds at the inferior ventral striatum, ventral rostral putamen, and dorsal caudal putamen^33^. If similar differences were observed with other seeds in the basal ganglia, then the differences in the DC FC were not specific. Conversely, if comparable connectivities between groups were observed within other seeds in the basal ganglia, then the results were strengthened, because they were specific to the DC.

### Statistical analyses

SPSS Statistics 20.0 (SPSS Inc., Chicago, IL) was used for the rest of the analyses. Results were considered significant at *p* < 0.05 (two-tailed). A two-sample *t*-test (or a Mann–Whitney *U* test, if the sample was not distributed in a Gaussian manner) was conducted to examine between-group (BD II patients vs. HC) differences in demographic characteristics, HDRS score, YMRS score, plasma level of OXT, and striatal DAT availability.

### Image analysis

A two-sample independent *t*-test was employed to analyze the FC maps using SPM 12 (Wellcome Trust Centre for Neuroimaging, London, https://www.fil.ion.ucl.ac.uk/spm/). Statistical maps were computed to identify changes in the DC-seeded FC for between-group comparisons. Significance was thresholded at the voxel-level family-wise error rate (FWE)-corrected *p* = 0.05 for whole-brain multiple comparisons.

Two two-sample *t*-tests were performed to determine the correlations between the DC-seeded FC and the OXT level or striatal DAT availability, respectively. We entered the demeaned (in SPM) values as a regressor to identify brain regions with either positive or negative correlations with the DC-seeded FC in the BD II patients or HC. Significance was thresholded at the uncorrected voxel-level *p* = 0.001, followed by the FWE-corrected cluster-level at *p* = 0.05.

To display 3D imaging, we used MRIcroGL for 3D rendering (Department of Psychology, University of South Carolina http://www.mccauslandcenter.sc.edu/mricrogl). To further show the regression results in scatterplots, we extracted the DC-seeded FC values in the PPC (peak MNI coordinates [–28, –48, 56] and [–24, –66, 58], radius = 6 mm). The corresponding correlation coefficients (*r*) and *p* values were analyzed using SPSS Statistics 20.0.

## Results

Twenty-five BD II patients and 29 HC were enrolled in this study. The BD II patients received mood stabilizer treatment, including valproic acid (*n* = 10, 40.0%), valproic acid plus antipsychotics (*n* = 10, 40.0%), and only antipsychotics (*n* = 3, 12.0%).

There were no significant differences between the BD II patients and HC in the demographic data (Table 1). The BD II patients in this study scored 5.29 ± 5.24 on the HDRS and 1.30 ± 2.03 on the YMRS, and 16 of them were euthymic (scores of <7 on the HDRS and YMRS; Table 1). The BD II patients, in comparison to the HC, demonstrated a higher YMRS score and a lower OXT level, but there was no significant difference in striatal DAT availability (Table 1).

**Table 1:** Demographic data and baseline information.

	BD II patients (*n* = 25)	Controls (*n* = 29)	*p* value
Age, years	36.08 ± 11.64	32.83 ± 10.96	0.400
Gender, female (%)	17 (68%)	17 (59%)	0.576
Body mass index^a^	27.52 ± 5.72	25.78 ± 5.85	0.281
Education, years^b^	14.20 ± 3.14	15.36 ± 2.02	0.273
HDRS score^c^	5.29 ± 5.24	2.92 ± 1.41	0.300
YMRS score^d^	1.30 ± 2.03	0.00 ± 0.00	<0.001*
Oxytocin (pg/mL)^e^	167.27 ± 130.21	248.45 ± 52.99	0.012*
DAT availability^f^	1.47 ± 0.22	1.43 ± 0.26	0.591

The data are presented as the means ± SD.

*p < 0.05.

^a^One bipolar disorder (BD) II patient did not have body weight and body height measurements and was excluded from this calculation.

^b^Four controls did not answer the years of education question and were excluded from this calculation.

^c^One BD II patient and 4 controls did not complete the 17-item Hamilton Depression Rating Scale (HDRS) and were excluded from this calculation.

^d^Two BD II patient did not complete the 11-item Young Mania Rating Scale (YMRS) and were excluded from this calculation.

^e^Four BD II patients and eight controls did not undergo plasma oxytocin level measurement and were excluded from this calculation.

^f^Two BD II patients did not undergo TRODAT and were excluded from this dopamine transporter (DAT) availability calculation.

**Fig. 1:**
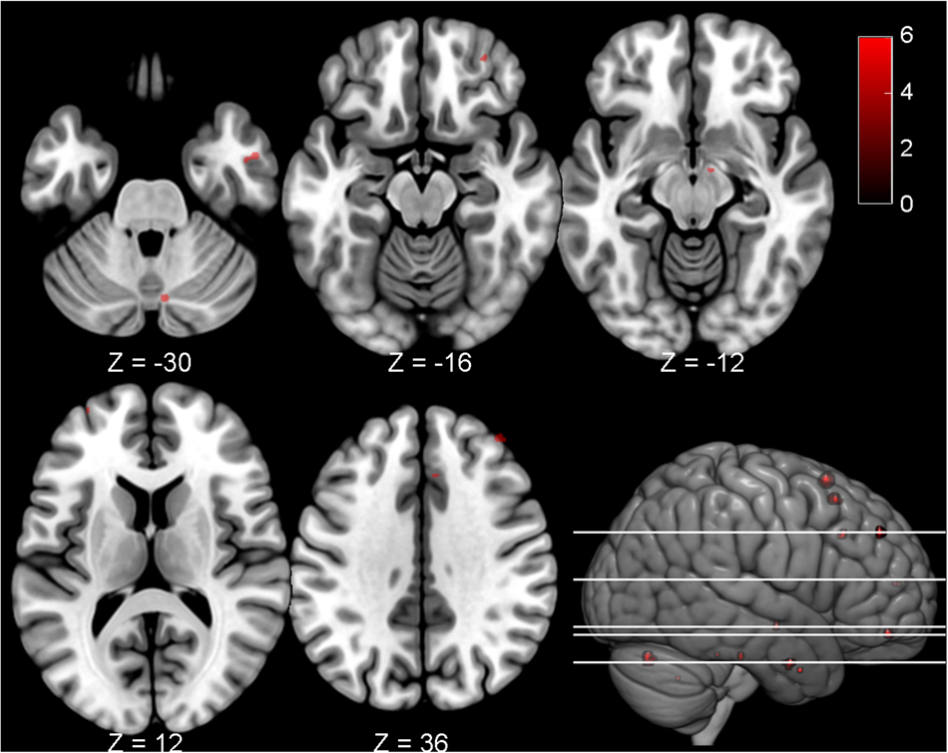
Regions showed significant functional connectivity with the left dorsal caudate in between-group comparisons comparing bipolar II disorder patients < healthy controls. The bipolar II disorder patients exhibited impaired functional connectivity between the left dorsal caudate and the pons, temporal, orbitofrontal, dorsolateral prefrontal, anterior cingulate cortex, supplementary motor area, and cerebellum as compared with the controls. Each region’s coordinates are listed in Table 2. Results were thresholded at the FWE-corrected voxel level *p* = 0.05. The color bar denotes the *t*-scores. Figures are displayed according to neurological convention (left = left).

The BD II patients exhibited significantly decreased FC between the left DC and the dlPFC, ACC, orbitofrontal cortex, temporal, supplementary motor area, pons, and cerebellum as compared with the HC (Fig. 1 and Table 2). Because the right DC-seeded FC analyses yielded similar results (i.e., DC-ECN hypo-connectivity, see Supplementary Table S1), we presented only the left DC-seeded FC maps in the following correlational analyses. In contrast, there were no significant group differences using seeds at the inferior ventral striatum, ventral rostral putamen, and dorsal caudal putamen as independent coordinates to validate our findings. These supplementary analyses supported the specificity of the DC-ECN circuitry in the BD II patients.

**Table 2:** Peak MNI coordinates for the regions exhibiting significant resting-state functional connectivity with the left dorsal caudate for between-group differences comparing BD II patients < healthy controls.

Region	Lateral	Cluster	BA	*t* score	Peak coordinate		
					*x*	*y*	*z*
Dorsolateral prefrontal cortex	R	7	9	6.40	40	46	36
Dorsolateral prefrontal cortex	L	2	10	5.61	–32	58	12
Supplementary motor area	R	20	6	6.26	6	18	64
Supplementary motor area	R	11	6	6.08	10	20	54
Middle temporal gyrus	R	9	21	6.05	52	–2	–30
Anterior cingulate cortex	R	4	32	5.87	6	26	34
Pons	R	3	—	5.79	10	–8	–12
Pons	R	2	—	5.75	8	–26	–28
Superior frontal gyrus	L	2	10	5.68	–20	52	10
Temporal pole	R	2	38	5.67	36	4	–34
Orbitofrontal cortex	R	5	11	5.66	32	48	–16
Cerebellum (Crus I)	R	12	—	6.05	8	–74	–28
Cerebellum (Hemisphere III)	L	1	—	5.54	–14	–38	–26
Cerebellum (Anterior lobe)	L	1	—	5.52	–20	–58	–38

Peak coordinates refer to the Montreal Neurological Institute (MNI) space. Significance was thresholded at the FWE-corrected voxel level *p* = 0.05.

No region was found in the contrast of bipolar disorder (BD) II patients > healthy controls.

*BA* Brodmann area.

**Fig. 2:**
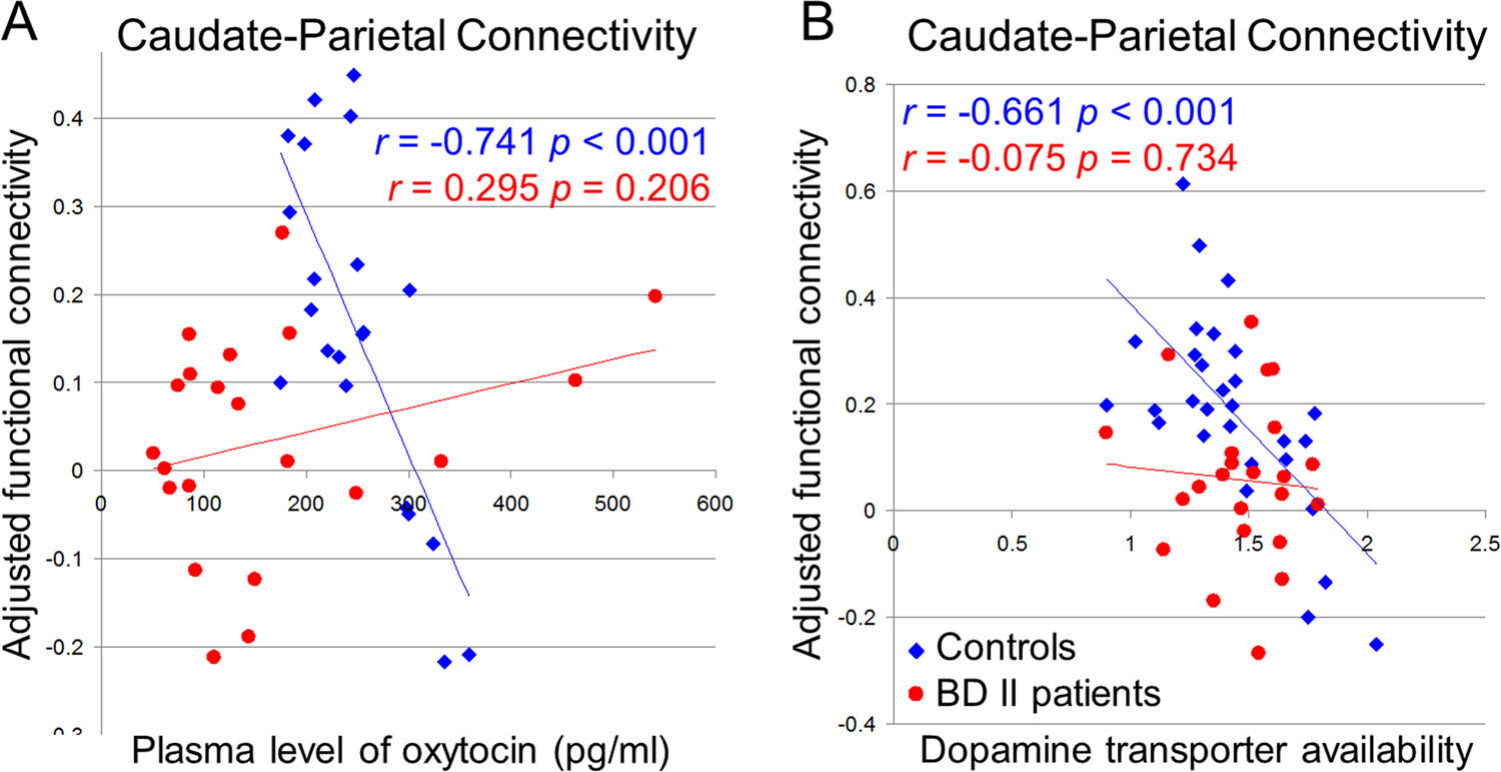
The caudate functional connectivity covaries with the oxytocin and dopamine transporter availabilities. The oxytocin and dopamine transporter (DAT) availabilities were correlated negatively with the dorsal caudate-posterior parietal cortex functional connectivity only in the healthy controls (blue diamond) and not in the bipolar II disorder (BD II) patients (red dot). The scatterplots disclose the relationships between the dorsal caudate-seeded functional connectivity around the peak voxel (see Table 3) and **a** oxytocin and **b** dopamine transporter (DAT) availability. The corresponding correlation coefficients (*r*) and *p* values are provided. Significance was thresholded at the uncorrected voxel level *p* = 0.001, followed by the FWE-corrected cluster level *p* = 0.05.

Among the HC, the DC-dlPFC and DC-PPC FC were negatively correlated with the plasma OXT level, while the DC-PPC FC was negatively correlated with the striatal DAT availability (Fig. 2 and Table 3). No correlation was found in the BD II patients.

**Table 3:** Functional connectivity of the left dorsal caudate co-varying with oxytocin and left striatal dopamine transporter levels in healthy controls.

						Peak coordinate		
Direction	Region	Lateral	Cluster	BA	*t* score	*x*	*y*	*z*
Oxytocin level^a^								
Negative	Posterior parietal cortex	L	6206	7	5.04	−28	−48	56
	Dorsolateral prefrontal cortex	L	141	10	3.95	−34	58	12
	Lingual gyrus	L	348	18	4.42	−14	−72	−4
	Lingual gyrus	R	298	19	4.06	16	−62	0
	Superior occipital gyrus	R	161	19	3.96	18	−82	32
Positive	Cerebellum (Crus II)	L	167	–	5.10	−32	−82	−42
Striatal DAT availability^b^								
Negative	Posterior parietal cortex	L	395	7	4.46	−24	−66	58
Positive	Cerebellum (Crus II)	L	763	–	5.77	−14	−88	−38
	Cerebellum (Crus II)	R	180	–	4.55	50	−60	−50

Peak coordinates refer to the Montreal Neurological Institute (MNI) space. Significance was thresholded at the uncorrected voxel level *p* = 0.001, followed by the FWE-corrected cluster level *p* = 0.05.

No correlation was found in the bipolar disorder (BD) II patients.

*BA* Brodmann area.

^**a**^Four BD II patients and eight controls did not have the plasma oxytocin level recorded and were excluded from this analysis.

^**b**^Two BD II patients did not undergo TRODAT and were excluded from this dopamine transporter (DAT) availability calculation.

## Discussion

Our results in the HC support a model in which the OXT and dopamine systems act in tandem to regulate the DC-ECN circuitry. However, such synergistic interaction was perturbed in the BD II patients, who had a lower OXT level and a lower DC-ECN hypo-connectivity as compared with the HC. Taken together, these results imply a maladaptive neuroplasticity in BD II. Also, our findings of alterations in FC between the DC and the orbitofrontal and anterior cingulate cortex were in line with the results of previous activity studies of BD^13,34^.

In the BD II patients, we found a dysfunctional DC-ECN connectivity (Fig. 1) and disrupted correlations of the DC-ECN connectivity with OXT and striatal DAT, implicating a perturbed reward system in BD II. Our reasoning was supported by the low OXT level in the BD II patients (Table 1). As OXT increases the FC between the corticostriatal circuitry^3^ and facilitates the DC-dlPFC loops for stable social bond formation^2^, the low OXT level in the BD II patients may be the reason for the dysfunctional corticostriatal circuitry, and may further result in poor social cognitive function^35^. Consistent with this observation, impaired ECN has also been found in premenstrual dysphoric disorder^36^ and repetitive negative thinking^37^, which are both related to altered reward systems (reinforced reward sensitivity^38^ and enhanced regret for no reward^39^, respectively).

In the HC, there were significant correlations between the DC-PPC FC and OXT level, as well as striatal DAT, suggesting oxytocinergic modulation of dopaminergic reward systems. Such OXT-driven dopaminergic modulation may have influenced social behaviors through control of motor activity in prior animal studies^1,40^. Consistent with previous findings, our research further demonstrated that the DC-PPC connectivity may be a fundamental neural signature in the corticostriatal circuitry. The DC-PPC connectivity has been reported in meta-analytic FC and diffusion tensor imaging studies^41^, and co-activation of DC-PPC is associated with decision-making^42–44^. Moreover, intranasal OXT may decrease dopamine release in the PPC and enhance attractiveness in a positron emission tomography (PET) study^45^. Given that the higher the OXT or DAT, the lower the DC-PPC connectivity (Fig. 2a, b), and the fact that the PPC is involved in spatial explorations^46,47^, our data provide a possible underlying mechanism of oxytocinergic modulation of dopaminergic reward systems dampening non-social exploration in the HC.

Our data showed hypo-connectivity between the DC and the cerebellum (Crus I) in the BD II patients (Table 1). In contrast, the DC-cerebellum (Crus II) FC was positively correlated with OXT and striatal DAT in the HC (Table 2). Both Crus I and Crus II may be involved in cognitive processing as they were more connected to the cortical ECN (dlPFC and PPC) during pain^48^. Furthermore, Crus I and Crus II are connected to the thalamus^48^, and altered cerebello-thalamo-cortical networks were found to be associated with psychosis^49^, and may be a heritable trait in schizophrenia^50^. Collectively, the DC-Crus II FC may interact with OXT and striatal DAT in HC, while the disrupted cerebello-DC-cortical circuitry may underlie the neuropathology of BD II.

### Limitations

Our study had limitations in the relatively small sample size. As a cross-sectional study, understanding consequential or causal roles among OXT, striatal DAT, and corticostriatal connectivity was difficult to affirm. OXT immunoreactivity levels cannot be used to accurately infer true values of OXT, and therefore cannot be compared between studies^51–53^. Nevertheless, it is not our purpose in the current imaging study to measure absolute values of OXT in the BD II patients; rather, we used these assays for the comparison of relative levels of peripheral OXT between groups^54^, as well as investigating the relationship between OXT levels and functional connectivity. Another concern was that the plasma OXT level may not predict the central concentration^51^; however, other imaging studies found that the plasma OXT level was positively correlated with left caudate activation, and may augment the reward systems^55^. We did not find alterations in the DMN FC and striatal DAT availability; nevertheless, stability of the DMN may reflect a state of remission in BD^56^. In the same way, the lack of between-group differences in striatal DAT availability may have also resulted from the fact that most of the BD II patients in this study were in a euthymic state (Table 1), and therefore had no impaired striatal activity.

## Conclusion

To the best of our knowledge, this was the first study to provide important and novel insights into the unexplored corticostriatal circuitry in BD II patients. The results extend existing knowledge from animal studies and offer some useful implications for neurobiological research going forward. The DC-PPC connectivity is a critical circuitry in the oxytocinergic modulation of dopaminergic reward systems, and dysfunctional DC-ECN circuitry implicates a maladaptive neuroplasticity in BD II patients, specifically. Other regions, such as cerebellum, are easily overlooked, but may also have important regulatory roles in the corticostriatal circuitry in BD II.

## Supplementary information

Supplemental Table S1
