# Supplementary material for: Dysregulation of oxytocin and dopamine in the corticostriatal circuitry in bipolar II disorder
Source: Transl Psychiatry. 2020 Aug 12;10:281. doi: 10.1038/s41398-020-00972-6 (PMC7423887; doi:10.1038/s41398-020-00972-6)
Supplement: Supplementary file 1 — Supplemental Table S1 [file 41398_2020_972_MOESM1_ESM.docx]

**Supplemental Table S1 Peak MNI coordinates for the regions exhibiting significant resting-state functional connectivity with the right dorsal caudate for between-group differences comparing BD II patients < healthy controls**

| Region | Lateral | Cluster | BA | *t* Score | Peak coordinate | | |
| --- | --- | --- | --- | --- | --- | --- | --- |
|  |  |  |  |  | *x* | *y* | *z* |
| Dorsolateral prefrontal cortex | R | 1 | 9 | 5.60 | 36 | 42 | 40 |
| Dorsolateral prefrontal cortex | R | 1 | 9 | 5.53 | 40 | 44 | 38 |
| Superior temporal gyrus | L | 7 | 41 | 6.31 | –68 | –24 | 8 |
| Inferior temporal gyrus | L | 1 | 20 | 5.53 | –46 | –24 | –26 |

Peak coordinates refer to the Montreal Neurological Institute (MNI) space.

Significance was thresholded at the FWE-corrected voxel level *p* = 0.05.

No region was found in the contrast of bipolar disorder (BD) II patients > healthy controls.

BA: Brodmann area.
